# A proof of concept phase II study with the PDE-4 inhibitor roflumilast in patients with mild cognitive impairment or mild Alzheimer’s disease dementia (ROMEMA): study protocol of a double-blind, randomized, placebo-controlled, between-subjects trial

**DOI:** 10.1186/s13063-024-08001-3

**Published:** 2024-03-04

**Authors:** Nina Possemis, Frans Verhey, Jos Prickaerts, Arjan Blokland, Inez Ramakers

**Affiliations:** 1 Dept. of Psychiatry and Neuropsychology, School for Mental, Health and Neuroscience, Alzheimer Centre Limburg, Maastricht University, Maastricht, The Netherlands; 2 Department of Psychiatry & Neuropsychology, School for Mental Health and Neuroscience, Maastricht University Medical Centre+ (MUMC+), Alzheimer Centre Limburg, Maastricht University, Maastricht, The Netherlands; 3 Department of Psychiatry and Neuropsychology, School for Mental Health and Neuroscience, Maastricht University, Maastricht, The Netherlands; 4 Faculty of Psychology and Neuroscience, Department of Neuropsychology & Psychopharmacology, EURON, Maastricht University, Maastricht, the Netherlands

**Keywords:** Mild cognitive impairment, Dementia, Alzheimer, Cognitive dysfunction, Roflumilast, Phosphodiesterase 4, Memory, Drug intervention, Randomized controlled trial

## Abstract

**Background:**

Research into the neurobiological underpinnings of learning and memory has demonstrated the cognitive-enhancing effects associated with diverse classes of phosphodiesterase (PDE) inhibitors. Specific PDE inhibitors have been identified to improve neuronal communication through selective inhibition of PDE activity. Roflumilast, a PDE4 inhibitor, has demonstrated efficacy in enhancing episodic memory in healthy adults and elderly participants with pronounced memory impairment, indicative of amnestic mild cognitive impairment (aMCI). In alignment with these findings, the present protocol aims to provide a proof of concept phase II of the potential of roflumilast to aid patients diagnosed with (a)MCI or mild Alzheimer’s disease (AD) dementia.

**Methods:**

The study will be conducted according to a double-blind, randomized placebo-controlled, between-subjects design. Participants with (a)MCI and mild AD dementia will be recruited through the Memory Clinic at the Maastricht University Medical Centre + (MUMC +) in Maastricht, the Netherlands, alongside outreach through regional hospitals, and social media. The study will have three arms: placebo, 50 μg roflumilast, and 100 μg roflumilast, with a treatment duration of 24 weeks. The primary outcome measure will focus on the assessment of episodic memory, as evaluated through participants’ performance on the 15-word Verbal Learning Task (VLT). Our secondary objectives are multifaceted, including an exploration of various cognitive domains. In addition, insights into the well-being and daily functioning of participants will be investigated through interviews with both the participants and their (informal) caregivers, we are interested in the well-being and daily functioning of the participants.

**Discussion:**

The outcomes of the present study aim to elucidate the significance of the PDE4 inhibition mechanism as a prospective therapeutic target for enhancing cognitive function in individuals with (a)MCI and mild AD dementia. Identifying positive effects within these patient cohorts could extend the relevance of this treatment to encompass a broader spectrum of neurological disorders.

**Trial registration:**

The Medical Ethics Committee of MUMC + granted ethics approval for the 4th version of the protocol on September 10th, 2020. The trial was registered at the European Drug Regulatory Affairs Clinical Trials (EudraCT) registered on the 19th of December 2019 (https://www.clinicaltrialsregister.eu/ctr-search/trial/2019-004959-36/NL) and ClinicalTrial.gov (NCT04658654, https://clinicaltrials.gov/study/NCT04658654?intr=roflumilast&cond=mci&rank=1) on the 8th of December 2020. The Central Committee on Research Involving Human Subjects (CCMO) granted approval on the 30th of September 2020.

## Administrative information

Note: the numbers in curly brackets in this protocol refer to the SPIRIT checklist item numbers. The order of the items has been modified to group similar items (see http://www.equator-network.org/reporting-guidelines/spirit-2013-statement-defining-standard-protocol-items-for-clinical-trials/).

**Table:** 

**Title {1}**	A proof of concept phase II study with the PDE-4 inhibitor roflumilast in patients with mild cognitive impairment or mild Alzheimer’s disease dementia (ROMEMA): study protocol of a double-blind, randomized, placebo-controlled, between-subjects trial
Trial registration {2a and 2b}	EudraCT number; 2019–004959-36 Protocol ID; NL72476.068.20 Clinicaltrials.gov; NCT04658654. Registered on December 8th, 2020
Protocol version {3}	Issue Date: 8th of December 2021 Protocol Amendment Number: 04
Funding {4}	Subsidizing party; ZonMw (The Netherlands Organization for Health Research and Development, Project number: 446002504).
Author details {5a}	Nina Possemis, MSc Alzheimer Centre Limburg, School for Mental Health and Neuroscience, Maastricht University, Dept. of Psychiatry and Neuropsychology Maastricht, The Netherlands Prof. Dr. Frans Verhey Alzheimer Centre Limburg, School for Mental Health and Neuroscience, Maastricht University Maastricht University Medical Centre + (MUMC +), Dept. of Psychiatry & Neuropsychology Maastricht, The Netherlands Dr. Jos Prickaerts School for Mental Health and Neuroscience, Department of Psychiatry and Neuropsychology, Maastricht University, Maastricht, The Netherlands Prof. Dr. Arjan Blokland Faculty of Psychology and Neuroscience, Department of Neuropsychology & Psychopharmacology. EURON, Maastricht University, Maastricht, the Netherlands Dr. Inez Ramakers Alzheimer Centre Limburg, School for Mental Health and Neuroscience, Maastricht University, Maastricht University Medical Centre + (MUMC +), Department of Psychiatry & Neuropsychology, Maastricht, The Netherlands
Name and contact information for the trial sponsor {5b}	Maastricht University / School of Mental Health and Neuroscience (MHeNs) Faculty of Health, Medicine, and Life Sciences (FHML) Universiteitssingel 50 6229 ER Maastricht The Netherlands
Role of study sponsor and funders {5c}	This study is subsidized by ZonMw (446,002,504). The funder provided marginal input during the review process of the study design through comments of the external reviewers of the original grant application. The sponsor (Maastricht University) developed the study design and is responsible for the collection, management, analysis, and interpretation of the data.

## Introduction

### Background and rationale {6a}

Society is faced with an increasing number of ageing people and, accordingly, concomitantly leading to a heightened prevalence of cognitive disorders and dementia, with Alzheimer’s disease (AD) emerging as the predominant etiological factor. Existing pharmacotherapeutic interventions targeting cognitive deficits in AD mainly include cholinesterase inhibitors, yet their efficacy remains limited [1]. Additionally, these drugs are associated with notable side effects such as, e.g. hallucinations, hypertension, and nausea [2, 3]. Unfortunately, extant research underscores the inadequacy of current pharmacological agents employed in the context of cognitive impairment related to an AD dementia diagnosis, if not even detrimental in individuals with mild cognitive impairment (MCI) [4, 5].

In the quest for innovative and effective pharmacotherapeutic interventions to aid cognitive deficits, preclinical studies have shown significant cognitive-enhancing effects of various phosphodiesterase (PDE) inhibitors [6]. These compounds improve neuronal communication by selectively inhibiting PDE activity, enzymes that inactivate the intracellular second messengers, namely cyclic adenosine monophosphate (cAMP) and cyclic guanosine monophosphate (cGMP) [7]. The underlying hypothesis posits that the facilitation of neuroplasticity through signal transduction stimulation may precipitate neuroprotective effects. Within the plethora of available PDE targets, this project is specifically centred on PDE4, a target identified as persistently present in the ageing brain and post-mortem brains of individuals diagnosed with AD dementia [8, 9]. Notably, PDE4 inhibitors have been demonstrated to improve cognitive function in several animal species, including executive functioning and planning in nonhuman primates [10, 11]. Moreover, these inhibitors have exhibited the capacity to augment brain plasticity and memory in rodent models of ageing and AD [6]. Intriguingly, in a preclinical mouse study focused on tauopathy, PDE4 inhibition was found to facilitate the clearance of aggregated tau, consequently improving cognitive performance in these mice [12].

The cognitive effects of PDE4 inhibitors have been systematically explored in healthy adult and elderly populations, revealing improvement in episodic memory and sensory gating [13, 14]. Nevertheless, the translational progress of PDE4 inhibitors into therapeutic drugs has been hampered due to dose-limiting emetic side effects. An example of such is the classic PDE4 inhibitor rolipram (Shering AG), originally developed as a potential antidepressant in the 1980s. Recent advancements in PDE4 inhibitor development have successfully mitigated emetic side effects, as exemplified by roflumilast (AstraZeneca). In 2010, roflumilast received approval as an anti-inflammatory medication, marketed as Daxas within the European Union, specifically for the treatment of chronic obstructive pulmonary disease (COPD) exacerbations [15]. In a pilot study, our department investigated elderly participants with pronounced age-associated memory impairment indicative of aMCI. Of note, these participants were recruited from the general population and had not been officially diagnosed by a physician. Further, the scope of these studies was confined to acute treatment/dose assessments. Importantly, favourable outcomes on memory were observed in both healthy elderly participants [16] and elderly participants with pronounced memory impairment, indicative of aMCI. The paradigm shift induced by the ineffectiveness of disease-modifying drug trials in AD treatment has redirected attention toward strategies aimed at delaying the progression from MCI to dementia. Focusing on the prodromal stage of AD holds promise for substantially mitigating the prevalence and associated costs of dementia. Given that MCI represents a possible transitional phase between normal ageing and dementia, its impact extends to 10–15% of the population aged over 65 [17].

The objective of the proposed proof-of-concept phase II study is to validate the potential cognitive-enhancing effects of chronic roflumilast treatment in individuals diagnosed with (a)MCI or mild AD dementia, spanning a 24-week duration. This investigative pursuit is a logical progression from antecedent studies conducted within our department, which demonstrated favourable outcomes following acute roflumilast treatment in healthy young, healthy elderly, and elderly participants with pronounced age-associated memory impairment indicative of aMCI. Noteworthy distinctions in the proposed study lie in its randomized controlled trial design encompassing participants diagnosed with (a)MCI or mild AD dementia, couples with the administration of roflumilast over a chronic timeframe. The emphasis on chronic dosing is grounded in the premise that sustained administration may yield cumulative neuroprotective effects, complementing the acute signal transduction stimulation observed in earlier investigations. In the event of a positive outcome, subsequent considerations may include the initiation of a multicentre phase III trial with patients diagnosed with MCI and/or early phase AD patients.

### Objectives {7}

This study aims to examine whether a 24-week administration of roflumilast improves cognition in participants with (a)MCI and/or participants with mild AD dementia. The outcomes may provide a proof of concept on the potential of roflumilast as a pharmacotherapeutic treatment to enhance cognition, concurrently advancing our comprehension of the role played by PDE4 in human cognition. The primary objective is to validate the effect of roflumilast on episodic memory, assessed with the 15-word verbal learning test (15-VLT), in aMCI/mild AD dementia participants after chronic roflumilast treatment of 24 weeks (i.e. 6 months). Specifically, we expect an increase in correct recall of words on the immediate and delayed recall. The 15-VLT is particularly relevant, as it assesses episodic memory, a function primarily associated with the hippocampus [18]. Considering the evidence of high PDE4 expression in this specific brain structure [19] and observed (early) atrophy of the medial temporal lobe regions in (a)MCI/mild AD [17, 20], we expect a larger effect size for cognitive functions and tasks related to this brain area. Moreover, Hamel and colleagues have demonstrated the prognostic value of the 15-VLT performance decline, predicting dementia approximately 7 years preceding the formal diagnosis [21]. This temporal insight underscores the test’s relevance and its potential to serve as a sensitive indicator of cognitive trajectories in the context of (a)MCI and mild AD.

Our secondary objectives encompass a comprehensive exploration of diverse cognitive domains. Included cognitive domains are as follows: mental speed/attention and executive functioning (Letter Digit Substitution Test (LDST) and Trail Making Test (TMT)), spatial memory (Spatial Pattern Separation Task), language (Alzheimer’s Disease Assessment Scale-Cognitive Subscale (ADAS-Cog), and the Shortened Boston Naming Test-15 (BNT-15)) and orientation (Mini-Mental State Examination (MMSE)) will be measured as well. In addition to cognitive assessments, we aim to gauge participants’ quality of life (Quality of Life AD (QoL-AD), EuroQol-5 Dimensions (EQ-5D)), psychological well-being (Hospital Anxiety Depression Scale; HADS), neuropsychiatric symptoms (Neuropsychiatric Inventory; NPI), and daily functioning (Alzheimer’s Disease Cooperative Activities of Daily Living Scale; ADCS-ADL). This multifaceted approach aims to illuminate the intricate interplay between cognition, quality of life, mood, and emotion. Outcome measures include a decrease in the reaction time (RT) in the LDST and TMT, as well as a decrease in errors in the LDST. Anticipated improvements encompass an increased total recognition score on the 15-VLT and an increased accuracy in all identified pattern completion scores of the pattern separation memory task. Total scores on the MMSE and ADAS-cog are expected to improve, as well as increased scores in well-being and quality of life, as indicated by the participant and the (informal) caregiver questionnaires. Furthermore, tau measurements from tear fluid samples will be assessed, as we speculate a potential positive impact of roflumilast on phosphorylated tau (pTau) concentrations [22]. Lastly, we will monitor the conversion from (a)MCI to dementia.

Three primary hypotheses have been formulated:

1. The administration of 50 μg roflumilast over a chronic treatment period of 24 weeks, compared to placebo, is anticipated to improve episodic memory in participants with aMCI or mild AD dementia;
2. The administration of 100 μg roflumilast over a chronic treatment period of 24 weeks, compared to placebo, is anticipated to improve episodic memory in participants with aMCI or mild AD dementia;
3. The administration of 50 μg roflumilast over a chronic treatment period of 24 weeks, compared to 100 μg roflumilast, is anticipated to have comparable effects on episodic memory in participants with aMCI or mild AD dementia.

### Trial design {8}

The ROMEMA study will be conducted according to a double-blind, randomized, placebo-controlled, superiority between-subjects design. The study includes a patient-based parallel group, with three arms (placebo, 50 μg roflumilast, and 100 μg roflumilast). Each arm comprises 27 participants, with randomization executed through block randomization, maintaining a balanced 1:1:1 allocation ratio.

## Methods: participants, interventions and outcomes

### Study setting {9}

All eligible patients diagnosed with (a)MCI and mild AD dementia will be invited to participate in the ROMEMA study. Participant recruitment will be exclusively conducted via the Memory Clinic of the Maastricht University Medical Centre (MUMC +) and outreach via social media channels. The ROMEMA study is explicitly designated as a mono-centre study. However, memory clinics of regional hospitals in the Netherlands and Belgium serve as additional recruiting centres for the sole purpose of raising awareness about the study, without active participation in the research procedures.

### Eligibility criteria {10}

To be eligible to participate in this study, prospective participants must meet the following eligibility criteria: age within the range of 50 and 90 years, willingness and capability of both the participant and (informal) caregiver, to provide and sign informed consent. Additional criteria include a body mass index (BMI) between 18.5 and 35, an MMSE total score of 20 or higher, and a clinical diagnosis of aMCI or mild dementia (probable AD) diagnosis, which entails a memory performance on the delayed recall of the 15-VLT of one or more standard deviation(s) below the normative score, corrected for age, education, and sex. Furthermore, a global clinical dementia rating (CDR) scale total score of 0.5 or 1. Mild dementia (probable AD) is defined according to the Clinical Core Criteria of McKahn in 1987, updated in 2011 [23].

Individuals undergoing current irradiation are excluded from the study as irradiation can cause radiation-induced cognitive impairment. Individuals who have a Fazekas score of 3 and higher on MRI scans are excluded as it indicates a mixed (AD and vascular) or vascular origin of cognitive impairment. Individuals with chronic viral infections (human immunodeficiency virus, and hepatitis B and C) are excluded from the study due to the possible risk of treatment-related toxicity. Normal pressure hydrocephalus (NPH), Morbus Huntington, Parkinson’s disease, recent transient ischemic attack (TIA), or cerebrovascular accident (CVA) within the last 2 years, TIA/CVA followed by a cognitive decline within 3 months are all exclusion criteria because of the cognitive impairment related to these disorders. COPD (gold criteria 3 and 4) are exclusion criteria due to the medication used in this study trial and its original indication. Roflumilast is a possible add-on treatment for COPD and severe asthma. Accordingly, although the dosage in this study is substantially lower than usually is prescribed in COPD patients (250 or 500 μg), we want to avoid cognitive improvement due to the improvement of any COPD-related symptoms. A lifetime history of schizophrenia, bipolar disorder, or psychotic symptoms not otherwise specified, current affective disorder (anxiety or major depression), cognitive problems due to alcohol abuse, brain tumours, epilepsy, and encephalitis are exclusion criteria because of the possible (temporary) cognitive impairment associated with these disorders. Other exclusion criteria are current treatment with (or other use of) cannabis, opiates, benzodiazepines, methylenedioxymethamphetamine, and cocaine. The use of medications strongly inhibiting CYP3A4 or CYP1A2 is also an exclusion criterion because of the interference with roflumilast metabolism resulting in the reduced therapeutic effectiveness of roflumilast. Individuals with rare hereditary problems of galactose intolerance, Lapp lactase deficiency, or glucose-galactose malabsorption will be excluded, as both the placebo and roflumilast contain lactose monohydrate. Moreover, participants are not allowed to participate in other drug trials during the study period. Lastly, participants unable to be accompanied at every test session by the same (informal) caregiver will be excluded. Roflumilast is contraindicated in individuals with moderate to severe liver impairment (e.g. Child–Pugh B and C) and those with hypersensitivity to the active substance or any of the listed excipients, namely, lactose monohydrate, corn starch, povidone, and magnesium stearate, will also be excluded from the study.

### Who will take informed consent? {26a}

Upon expression of interest and potential eligibility, the attending clinician will ask the patient for informed consent to be contacted by a researcher involved in the study. The researcher will elucidate the intricacies of the trial to the patient, providing a comprehensive understanding of its scope and implications. Should the patient maintain interest in participation, a patient information sheet detailing the study will be sent to the patient and their (informal) caregiver. The patient and the (informal) caregiver will be given at least 7 days to consider their interest and participation. If, following the contemplative period, both the patient and (informal) caregiver express sustained interest, they will be invited for the first visit (screening visit). During this visit, the researcher will address any lingering questions from the patient and (informal) caregiver. Following, the researcher, patient, and (informal) caregiver sign the informed consent. A physical copy of the signed informed consent is handed back to the participant and the (informal) caregiver.

### Additional consent provisions for collection and use of participant data and biological specimens {26b}

One additional blood sample of 9 ml will be collected during the screening visit for collaboration with other researchers of the Department of Pharmacology and Personalized Medicine of Maastricht University. This collaborative study will investigate the predictive potential of cAMP signal transduction preceding roflumilast administration, evaluating its correlation with drug response in participants. Moreover, the study aims to validate a precision diagnostic approach to endophenotype individuals with (a)MCI/mild AD dementia based on the state of cAMP, cGMP, and reactive oxygen species (ROS) signalling pathways. Material consent will be obtained to explicitly address the collection of this plasma specimen. Additionally, one blood sample of 9 ml will be collected during both the screening and the 24-week follow-up for collaboration with a researcher at the Department of Psychiatry and Neuropsychology of Maastricht University. This collaboration study will investigate and identify AD-specific molecular signatures through the analysis of the neuron-derived exosome (NDE) transcriptome. Furthermore, the study will engage in computational drug repurposing utilizing neuron-specific molecular signatures (exosomes). Material consent will be obtained to explicitly address the collection of these plasma specimens. Participants reserve the right to decline participation in both collaboration studies without any impact on their participation in the ROMEMA trial. Declining participation in the collaborative studies does not exclude them from the ROMEMA trial.

Supplementary consent will be obtained to establish the possibility of approaching participants for subsequent follow-up research, as well as to store biological samples for up to 5 years to use for additional research. Furthermore, participants will be asked for additional consent for the permission to request medical information from the participant’s general practitioner or treating specialist and permission to view the participant’s electronic patient record (if the participant is a patient at the MUMC +) for necessary information. Lastly, consent will be sought to inform the participant following unblinding (at the study end) of the study arm he/she was in. All additional consent obtained is explained in detail in the participant information sheet for clarity and transparency.

## Interventions

### Explanation for the choice of dosage comparators {6b}

To obtain insight into the optimal dosages concerning cognitive effects, this study will explore two distinct dosage levels of roflumilast, namely 50 and 100 µg, in addition to a placebo condition. The therapeutically recommended dosage for roflumilast in COPD is 500 µg. Our previous studies have investigated dose ranges up to 1000 µg to identify suitable dose levels for cognition enhancement. The recommended COPD dose of 500 µg results in similar plasma levels after chronic treatment as those after a single administration of the 1000 µg dose. Relatedly, to our knowledge, cognitive improvements in COPD patients have not been documented in the literature. A prior study from our department revealed that increased dosages beyond 100 µg did not yield a proportional increase in cognitive effects and, instead, resulted in adverse effects such as nausea and diarrhoea [24]. Building on these findings, it has been established that roflumilast at doses ranging from 5 times lower than the daily prescribed COPD dose of 500 µg, results in cognitively effective acute effects at a clinically meaningful degree [16]. Given the frequently observed inverted U-shaped dose–response curve in psychopharmacology, it is plausible that an even lower dose, such as 50 µg, may achieve comparable effectiveness when administered chronically to attain similar plasma levels. In addition, neuroprotective effects are expected to add up to the acute signal transduction simulation.

### Intervention description {11a}

Roflumilast, known by its trade names Daxas (EU) and Daliresp (US), is a non-steroidal anti-inflammatory drug designed to target pulmonary inflammation. The chemical nomenclature of roflumilast is delineated as N-(3,5-dichloropyridin-4-yl)-3-cyclopropylmethoxy-4-difluoromethoxy-benzamide. Its mechanism of action revolves around the reversible, selective inhibition of PDE4, resulting in the accumulation of intracellular cAMP levels. Following a single 500 µg dose, peak plasma concentrations are achieved approximately 60 min post-administration, with a range extending from 0.5 to 2 h. The terminal half-life is approximately 17 h, while the major pharmacodynamically active metabolite, roflumilast N-oxide, exhibits a half-life of 30 h. Elimination pathways involve approximately 20% excretion and 70% urine as inactive metabolites. The pharmacokinetics of both roflumilast and its N-oxide metabolite demonstrate dose-proportional behaviour across a dosage spectrum spanning from 250 µg to 1000 µg. Presenting itself as a D-shaped film-coated tablet at a dose of 500 µg, this formulation is not suitable for the current application. In its standard form, roflumilast is a white to off-white non-hygroscopic powder, characterized by a melting point of 160 °C. Furthermore, the compound exhibits practical insolubility in water and hexane.

Basic Pharma, a good manufacturing practice (GMP) qualified company, has been appointed to order and reprocess roflumilast tablets into capsules tailored to the specific doses required for the study. The tablets will be crushed to a powder, which will then be blended with filler lactose monohydrate in the appropriate proportions. Each roflumilast capsule contains the following inactive ingredients: lactose monohydrate, corn starch, povidone, and magnesium stearate. The placebo capsule exclusively contains the principal constituent, lactose monohydrate. Capsules of size 0 will be manufactured with 0 µg (placebo), 50 µg, and 100 µg roflumilast doses. An independent researcher will provide Basic Pharma with a randomization list, and the labelling of capsule jars will be performed by Basic Pharma to uphold the double-blind study design. For each participant, Basic Pharma is instructed to prepare 186 pills distributed across two jars. The shipment of the study medication to the MUMC + trial pharmacy will be handled by Basic Pharma in multiple batches. The MUMC + trial pharmacy will oversee the storage of the capsules, conducting a comprehensive evaluation of each bath upon delivery and an inventory form will be signed in case of a positive evaluation of the batch. Researchers who are part of the study will be authorized to collect the jars with the study medication using a prescription. Participants will receive a jar containing 93 pills for the following 3 months. Upon the 12-week test day, participants will receive the second and final jar of 93 pills for the subsequent 3 months. An independent researcher will provide the MUMC + trial pharmacy with the randomization list in advance, ensuring the continued blinding of jars, participants, and involved researchers. The MUMC + trial pharmacy has been appointed for the responsibility of drug accountability, undertaking pill count, disposal, and proper storage of the medication. Participants will be instructed to take one capsule daily around the same time with water, with the flexibility to consume it with or without food. There are no further dietary restrictions, except for the prohibition of alcohol consumption (24 h before testing) and no smoking or the intake of caffeinated drinks in the morning before test sessions.

### Criteria for discontinuing or modifying allocated interventions {11b}

Participants are free to withdraw their consent to participate at any time for any reason if they wish to do so, without any consequences or need for an explanation. The investigator holds the prerogative to terminate a participant’s participation in the study in instances of non-compliance, such as medication adherence. In situations where participants encounter side effects resulting in substantial harm or rendering continued participation unfeasible, the investigator may opt to remove the participant from the study, particularly in the event of a serious adverse event [25].

### Strategies to improve adherence to interventions {11c}

A systematic review of medication non-adherence in individuals with cognitive impairment underscores the susceptibility of elderly individuals diagnosed with MCI and mild dementia to non-adherence, given the cognitive demands inherent in medication management [26]. Accordingly, we will incorporate a study medication diary as an intervention to enhance medication adherence. The diary will inform them about optimal storage and administration practices for the study medication, along with guidance for instances where participants have forgotten to take a capsule. Participants will be required to record daily whether they have adhered to the medication schedule and at what specific time. Additional space has been added for participants to document any particularities, comments, side effects, or other noteworthy observations. Note that the study medication diary answers are not an outcome of the study and thus will not be analyzed. To further increase medication adherence, participants will receive a pill box to facilitate the organized distribution of capsules throughout the week. The provision of additional capsules will serve as a contingency for unforeseen accidents, such as dropping a capsule in the sink, while also serving as a metric for evaluating medication adherence. Unused capsules and bottles will be collected during the 12-week and 24-week visits, with the trial pharmacy documenting and quantifying any surplus study medication. An effort will be made by the researcher to involve a committed (informal) caregiver of the participant to monitor medication adherence. Additionally, regular bi-weekly calls from the research team will provide a platform for participants to discuss and address any challenges or concerns related to the study medication and medication adherence.

### Relevant concomitant care permitted or prohibited during the trial {11d}

All concomitant care, except treatment with opiates and benzodiazepines, is permitted during the trial. Treatment with opiates and benzodiazepines is known to affect memory, and thus anyone using opiates and/or benzodiazepines will be excluded from the study. Comprehensive documentation of all other concomitant care will be recorded in the electronic case report form (eCRF). Participants are expected to adhere to their regular medication regimens for the management of pre-existing conditions throughout the trial period.

### Provisions for post-trial care {30}

Participants are informed in the participant information sheet that insurance is provided for everyone participating in this study. The insurance covers damages/harm caused by the study; however, not all damages/harm are covered. In the participation information sheet, we inform participants that insurance does not apply if the risk occurs more seriously than was foreseen or if the risk is very unlikely; harm to his/her health that would also have occurred if they had not participated in this study; harm to their result, as a result of a negative effect of the study on them or their children and damage due to an existing treatment method in the case of existing treatment methods. The insurance provides coverage of at least 650,000 euros per participant and at least 5,000,000 euros for the entire study. The insurance applies to harm during the study or within 4 years of the end of the participation in the study. These provisions can be found in the “Decree on Compulsory Insurance in Medical Scientific Research with Humans 2015.” This decree can be found in the Government Law Database (https://wetten.overheid.nl). Participants are informed in the participant information sheet that the study medication is not distributed post-trial participation.

### Outcomes {12}

#### Primary study parameter

The primary outcome measure is verbal memory using the 15-VLT [27]. The 15-VLT is chosen as our primary outcome because it assesses episodic memory—a cognitive domain linked with hippocampal function [18]. Considering the evidence of high PDE4 expression in the hippocampus [19] and observable atrophy in the medial temporal lobe regions of individuals diagnosed with MCI [17, 20]. Accordingly, a larger effect size for cognitive functions and tasks related to this brain area is expected. Variables include correct immediate recall on trial 1 to trial 5, correct total immediate recall (sum of all 5 trials), and correct delayed recall. Change from baseline to acute effects, change from baseline to 3 months, change from baseline to 6 months, and 6 months to follow-up will be statistically analysed.

#### Secondary study parameters

Our secondary objectives include a comprehensive evaluation of various cognitive domains, extending beyond the primary focus on verbal memory. The LDST serves as a metric for assessing visual scanning, mental flexibility, sustained attention, psychomotor speed, and speed of information processing. The LDST outcomes include the total number of correct written items after 90 s, and the total number of correct items read after 90 s. The TMT measures visual attention (Part A) executive functioning and mental flexibility (Part B). The TMT outcome is the time (in seconds) required to complete each part individually (Part A and Part B). Spatial memory is measured by the spatial pattern separation task, evaluating participants’ accuracy in identifying the position of objects on a computer screen. The ADAS-Cog will be used as it measures language and memory in general. Subtests within the ADAS-Cog encompass naming objects and fingers, following commands, constructional praxis, ideational praxis, orientation, remembering test directions, spoken language, comprehension, and word-finding difficulty. The total score of the subtests is utilized as the ADAS-Cog. The word recall task and the word recognition task have been removed from the task, to avoid interference with our primary outcome measure (15-VLT). The 15-VLT recognition task is part of the secondary outcomes. The MMSE serves as a screening tool for general cognitive impairment, with the total score acting as the MMSE outcome. Confrontational naming is assessed through the Shortened BNT-15, with the total score derived from correctly named items, either spontaneously or after semantic cues.

Furthermore, our secondary objectives extend beyond cognitive assessments to include well-being and quality of life questionnaires for both participants (QoL-AD, EQ-5D, HADS) and their (informal) caregiver (NPI, ADCS-ADL). The outcome measure of the EuroQol, the QoL-AD, and the ADCS-ADL is the total score. The outcome measure of the HADS is the total score, as well as sub-scores for depression and anxiety separately. The outcome of the NPI is the total care score (frequency × severity) and total burden score. Suicidal intent and thoughts about suicide are explored using item 9 of the Beck Depression Inventory 2 (BDI-2). For all secondary outcomes, the change from baseline to acute effects, change from baseline to 3 months, change from baseline to 6 months, and 6 months to follow-up will be statistically analysed.

#### Other outcomes

We aim to conduct a pharmacokinetic validation of roflumilast and its active metabolite roflumilast N-Oxide in blood plasma at T1 (acute test day), T2 (3-month test day), and T3 (6-month test day). As this is the first study to investigate a dose of 50 μg of roflumilast, a previous pharmacokinetic validation has never been performed for this dose specifically. Considering the inverted U-shaped dose–response curve, we expect 50 μg to reach the same effectiveness as 100 μg by likely achieving similar plasma levels after chronic administration. The outcome should determine approximately the same plasma level of roflumilast and its active metabolite per participant at measurements T2 (3 months) and T3 (6 months) if the participant complied with study medication adherence. We expect participants who were allocated in the 50 μg condition to have lower plasma levels than participants who were allocated in the 100 μg condition at the acute measurement. At the beginning of participation, participants will be required to complete a concise questionnaire, capturing essential demographic and lifestyle variables. This includes the date of birth, age, sex, highest education completed, civic status, living situation, housing situation, ethnicity, height, weight, amount of smoking per day, average alcohol intake per week, relationship with the (informal) caregiver, and any positive family history pertaining dementia, Parkinson’s disease, or amyotrophic lateral sclerosis. Upon study completion, a follow-up assessment will be conducted to identify any changes in the aforementioned variables. In addition to these demographic aspects, tau in tear fluid will be measured. Based on the current literature, it is possible to measure tau in tear fluid [22]. Our study aims to discern whether roflumilast induces changes in tau concentrations within tear fluid. The statistical analysis will focus on changes from baseline to the 6-month mark. Furthermore, vigilant monitoring of adverse events (AEs) and serious adverse events [25] will be integral to our study.

### Participant timeline {13}

**Fig. 1:**
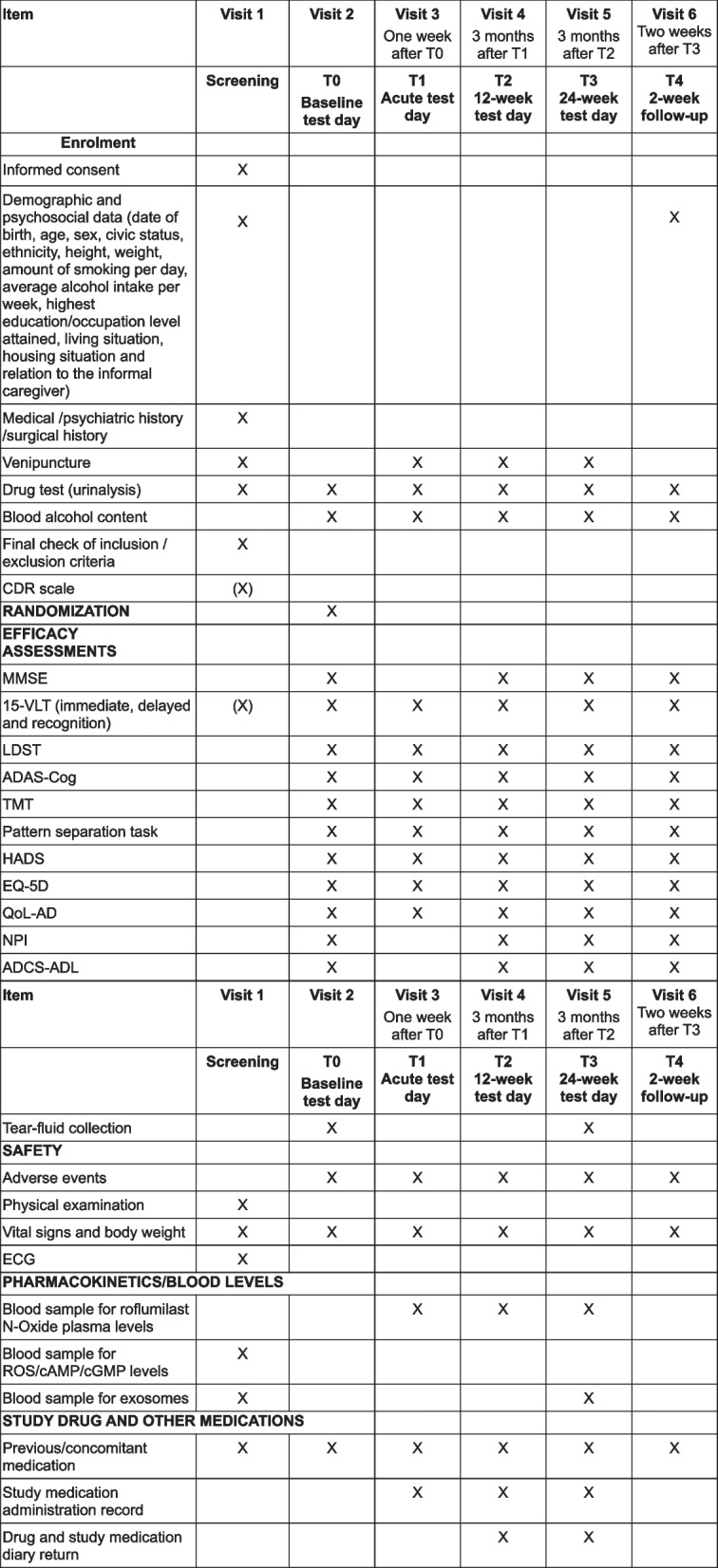
Trial schedule. MMSE: Mini-Mental State Examination, 15-VLT: 15-word Verbal Learning Task, CDR: Clinical Dementia Rating, LDST: Letter Digit Substitution Task, ADAS-Cog: Alzheimer’s Disease Assessment Scale – Cognitive subscale, TMT: Trail Making Test, HADS: Hospital Anxiety Depression Scale, EQ-5D: EuroQol – 5 Dimensions, QoL-AD: Quality of Life Alzheimer’s Disease, NPI: Neuropsychiatric Inventory, ADCS-ADL: Alzheimer’s Disease Cooperative Study—Activities of Daily Living, ECG: Electrocardiogram, ROS: reactive oxygen species

**Fig. 2:**
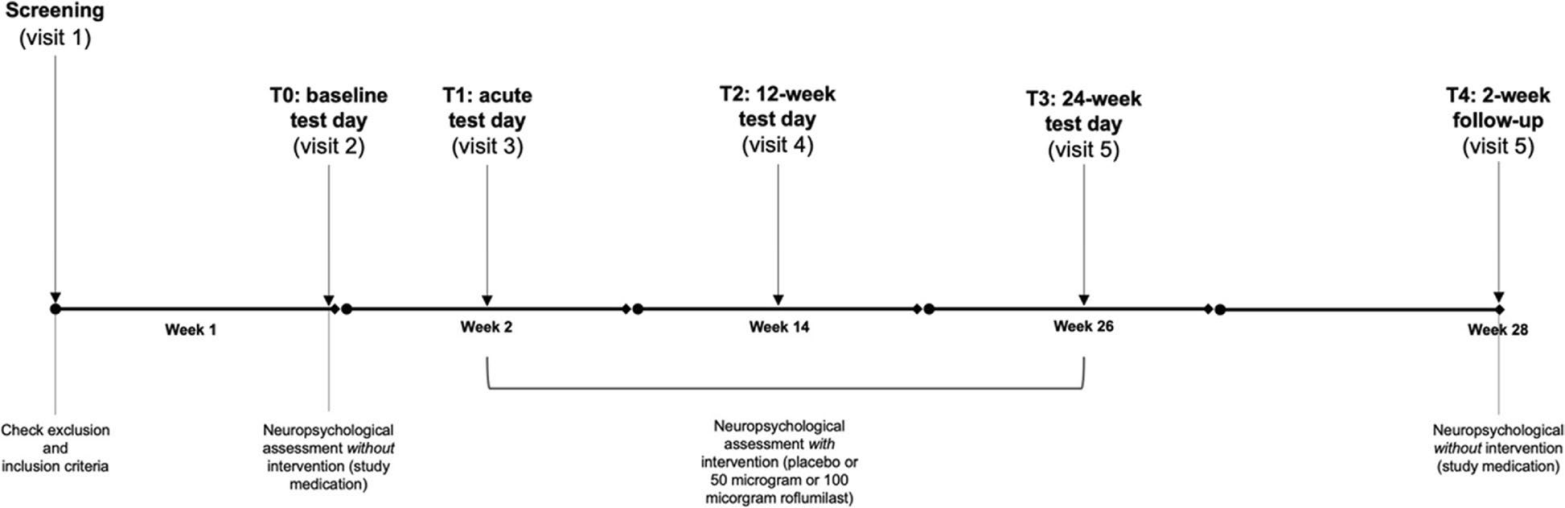
Schematic overview of test days

The current study consists of a medical screening, a baseline assessment, and subsequent acute, 12-week, 24-week, and 26-week follow-up test sessions. Figure 1 provides a detailed schedule of enrolment, interventions, and assessments. Preceding the medical screening, participants will receive a standardized medical questionnaire for completion at home. The voluntary submission of this questionnaire is encouraged to expedite the screening process. This questionnaire, solely intended for the research physician, will aid in determining eligibility criteria and does not constitute an outcome measure subject to statistical analysis. The screening will start with an explanation of the study, followed by signing the informed consent by the patient, the (informal) caregiver, and the researcher of the study. Subsequently, the MMSE, integral to inclusion criteria, will be administered. The medical screening will be performed by a research physician familiar with the study’s eligibility criteria. The first part of the medical screening involves an inquiry into the participants’ medical history through the medical questionnaire. Following, a physical examination will be performed, including checking appearance, extremities, skin, head, neck, eyes, ears, nose, throat, lungs, chest, heart, abdomen, and musculoskeletal system. Measurement of weight, height, systolic/diastolic blood pressure, and pulse rate will follow 5 min of rest in a supine position. Additionally, a resting 12-lead electrocardiogram will be recorded at a paper speed of 25 mm/s with at least 3 valuable beats per lead and a standard calibration (implying 1 mV = 10 mm for each lead). Blood samples (12 ml) will be collected for haematological and biochemical analyses. Urine samples will be processed for examination of urine status, urine sediment on indication, as well as a drug screening for cocaine, amphetamine, methamphetamine, marihuana, methadone, MDMA, morphine, barbiturate, and benzodiazepines. In instances where the 15-VLT and CDR scale data are unavailable, an assessment of these parameters will be conducted as part of the inclusion criteria. Participants with positive screening results will be invited for the baseline assessment. All visits have to be no more than 3 days after or before the set dates. Refer to Figs. 1 and 2 for a comprehensive overview of the study timeline and respective test days.

### Sample size {14}

Following a previous study demonstrating an estimated effect size of 0.69 (Cohen’s *d*) in healthy elderly and individuals with pronounced age-associated memory impairment [16], a power calculation for *F*-tests has been performed. This results in group sizes of 24 participants per group (Power 0.8; alpha 0.05), in consideration of potential dropout rates observed in the previous study, wherein nearly 10% of the participants withdrew for reasons unrelated to drug effects. To mitigate potential attrition, an additional 3 participants per treatment group will be included, resulting in 27 participants per group.

### Recruitment {15}

The recruitment strategy for this study involves a multifaceted approach. Primarily, participants will be recruited via the Memory Clinic of the MUMC + , targeting patients diagnosed with (a)MCI or mild dementia, along with their (informal) caregivers. Treating doctors at the Memory Clinic will assess eligibility using an exclusion checklist provided by the study investigators. While the study is designated as a mono-centre, collaboration with regional hospitals in the Netherlands and Belgium, such as Zuyderland Medical Centre, will be instrumental in extending outreach. Treating doctors at these regional hospitals will be informed about the study, allowing them to provide eligible patients with the patient information sheet. Additionally, recruitment efforts will extend to online platforms and relevant websites including those of Alzheimer Nederland (www.alzheimer-nederland.nl), Alzheimer Centrum Limburg (www.alzheimercentrumlimburg.nl), and Hersenstichting (www.hersenstichting.nl). Thematic newsletters and social media channels will also be leveraged. Prospective participants expressing interest will be guided by the researcher provided with the patient information sheet and granted a minimum of 7 days for thoughtful consideration. To validate the specific diagnosis, patients will be requested to bring the confirmation letter of their diagnosis from their medical specialist [28] to the screening visit, ensuring precision in participant selection.

## Assignment of interventions: allocation

### Sequence generation {16a}

Randomization and treatment allocation will be performed by an independent researcher, as the trial will be conducted in a randomized double-blind manner. The allocation sequence will be generated by an independent researcher using a computerized random number generator (https://commentpicker.com/random-number-generator.php). The randomization will be a block randomization of 12 participants (randomization within these blocks). The randomization list will be sent to the GMP-certified manufacturer and the trial pharmacy of the MUMC + .

### Concealment mechanism {16b}

The independent researcher will be responsible for conveying the randomization list to the GMP-certified manufacturer. Employing a coding system, the manufacturer will assign codes to the various treatments, facilitating the blinding of the study medication. The manufacturer will then proceed to prepare coded jars containing the study medication, adhering to the predetermined dosage levels. The manufactured and coded study medication will undergo a comprehensive quality control process to ensure adherence to regulatory standards. Subsequently, the coded jars will be shipped to the trial pharmacy of the hospital. A researcher of the study will be able to pick up the study medication based on a prescription from a medical doctor.

### Implementation {16c}

The randomization list will be sent to the manufacturer and the trial pharmacy. Subsequently, the researcher will oversee the enrollment of participants, who will be systematically allocated to specific interventions based on their designated participant numbers. This method ensures an unbiased and randomized assignment, contributing to the scientific rigour and validity of the study design.

## Assignment of interventions: blinding

### Who will be blinded {17a}

Trial participants, their (informal) caregivers, care providers, and the research team will remain blinded throughout the research. Only the independent researcher taking care of the randomization and concealed treatment allocation sequencing, the manufacturer that will produce the study medication, and the MUMC + trial pharmacy will know the participant’s treatment allocation. The research physician will be employed as a backup to unblind in case the independent researcher is not available in case of urgency. The study medication will be produced in identically appearing capsules in identically appearing jars, with neutral identification capsule jar labelling, such as 01(subject)_1(jar) for the first 12 weeks and 01(subject)_2(jar) for the subsequent 12 weeks.

### Procedure for unblinding if needed {17b}

In the case of unblinding, the research team has appointed an independent researcher to unblind. In situations where the designated independent researcher is unavailable, the research physician associated with the study is authorized to perform the unblinding process. Unblinding is a carefully regulated procedure and is only permissible under specific circumstances, particularly in emergencies such as (S)AEs, where a care provider requires knowledge of the administered dosage of the study medication.

## Data collection and management

### Plans for assessment and collection of outcomes {18a}

#### Verbal learning task (VLT)

In this study, the Dutch 15-VLT of Brand and Jolles (1985) serves as the primary tool for evaluating episodic memory [29]. This test entails a sequence of 15 Dutch monosyllabic words, presented for 1 s each, with an inter-stimulus interval of 1 s. Participants undergo five consecutive trials, with each trial culminating in a free recall of the presented words. The sum of the five recall trials results in the immediate recall score. Following a 20-min delay, during which no verbal memory tasks are performed, the participants are unexpectedly prompted to recall the previously learned words (delayed recall). Subsequently, a recognition test is administered, involving the discrimination of 15 stimulus words from 15 distractor words in a yes/no format. To mitigate learning effects, five Dutch parallel versions of the 15-VLT are utilized. Key dependent measures are the total number of correctly remembered words in the five learning trials (total immediate recall score), reflecting short-term verbal declarative memory, learning, and retrieval. Furthermore, the delayed recall score assesses the number of correctly recalled words after the 20-min interval. The amount of correctly recognized words assesses long-term verbal memory retrieval. The 15-VLT has well-established normative data derived from a cohort of over 1800 healthy individuals in the Dutch population [27].

#### Mini-mental state examination (MMSE)

The Dutch standardized MMSE is a commonly used screening instrument for cognitive function [30] consisting of 13 items that evaluate the domains of orientation, registration (immediate memory), short-term memory, attention, and language functioning. The maximum score is 30. The outcome measure of the MMSE is the total score. The instrument demonstrates commendable test–retest reliability and exhibits acceptable sensitivity and specificity, rendering it proficient in detecting mild to moderate stages of dementia [31].

#### Trail making test (TMT)

The TMT is a neuropsychological test designed to measure visual attention and task-switching capabilities [32]. The TMT provides information about visual search speed, scanning efficiency, processing speed, mental flexibility, and executive functioning. Compromising Part A and Part B, Part A instructs participants to connect a set of 25 circles—including the numbers 1 to 25—in sequential order as quickly as possible while still maintaining accuracy. Part A is used to primarily evaluate cognitive processing speed. Meanwhile, Part B evaluates executive functioning, specifically mental flexibility, as participants connect numbers and letters alternately (1, A, 2, B, etc.). The primary performance metric is the time in seconds taken to complete each part of the test individually, with the assumption that any errors made will be reflected in the completion time.

#### Letter digit substitution test [33]

The LDST [34], an adapted version of earlier substitution tests such as the Digit Symbol Substitution Test, serves as a tool for assessing information processing speed. Participants are presented with a test sheet featuring a key on the top, associating numbers 1 to 9 with different letters. The test items, displayed beneath the key, require participants to replace randomized letters with the corresponding digit indicated by the key. To familiarize participants with the task, initial 10 items serve as a practice round. Following these, participants are instructed to complete the remaining items as quickly as possible, by writing them down in the first round and naming them out loud in the second round. The dependent variables are the number of correct substitutions 90 s for both writing and naming rounds.

#### Boston naming test, 15 items (BNT-15)

The BNT-15 [35], as outlined by Kaplan et al., serves as an instrument for evaluating language performance. Participants are tasked with naming a series of objects within a stipulated timeframe of 20 s. The test compromises five easy, five moderately difficult, and five difficult items. The total score is derived from items correctly named spontaneously, plus additional items named correctly after semantic cues.

#### Alzheimer’s disease assessment scale – cognitive subscale (ADAS-Cog)

Developed in a collaborated effort in the 1980s, the ADAS-Cog is seen as a widely used cognitive assessment in clinical trials to measure the efficacy of pharmaceutical interventions regarding dementia [36, 37]. The ADAS-Cog includes participant-completed and observer-based assessments: summarized, word recall, naming objects and fingers, commands, constructional praxis, ideational praxis, orientation, word recognition, and language. Language includes the assessment of quality of speech, as well as comprehension of spoken language. For our study, we have removed the word recall and word recognition because of interference with the 15-VLT. It is important to mention that some significant variance in administration and thus reliability has been found [36]. Subsequently, it is imperative to ensure that personnel involved in the administration of ADAS-Cog undergo thorough training in adherence to standardized operating procedures.

#### Spatial pattern separation memory test

The pattern separation memory test [38] is a computer-based assessment designed to evaluate episodic memory, utilizing a collection of 140 colour images depicting commonplace neutral objects against a white background. This cognitive task consists of two phases. The first phase is the encoding phase. During this initial stage, participants are presented with each image and tasked with categorizing it as either “nice” or “not nice” by pressing designated buttons. Each image is displayed for 2 s, with a 0.5-s inter-stimulus interval. Immediately following the encoding phase, participants are informed of a surprise recognition memory test, which is the test phase. In this phase, participants are required to accurately discern whether images appeared in the same position on the screen as during the encoding phase. Notably, 40 images maintain their original position, while the remaining images are presented in varying positions on the screen, ranging from close to more distant (four different distances, 20 pictures each). Participants indicate their responses by pressing a designated key for the same position and a different designated key for a different position.

#### Hospital anxiety and depression scale (HADS)

The HADS [39] serves as a comprehensive tool for assessing the participant’s level of anxiety and depressive symptomatology. This 14-item scale is divided into two subscales, with 7 items addressing anxiety-related symptoms, and the remaining 7 items focusing on symptoms associated with depression. Each item on the questionnaire is assigned a score, with six items scored positively on a scale ranging from 0 to 3, and eight items scored inversely from 3 to 0. Consequently, the total score for each subscale falls within the range of 0 to 21, providing a quantitative representation of anxiety and depression levels. Classification of participants is determined based on predefined cutoff points: “non-cases” (total score of 7 or less), “doubtful cases” (total score between 8 and 10), and “definite cases” (total score of 11 or higher). The established cutoff point of 8 out of 21 for anxiety or depression, as derived from the work of Bjelland and colleagues, adds consistency to the interpretation of results. The HADS has demonstrated good overall reliability, as well as good validity [40].

#### EuroQol quality of life (EQ-5D)

The EQ-5D [41] is an instrument that evaluates the quality of life, originating in the European context. It is a preference-based health-related quality-of-life measure with one question for each of the five dimensions including mobility, self-care, usual activities, pain/discomfort, and anxiety/depression. A weighted health index can be derived from this for an individual. In addition, participants will rate their general health status on a visual analogue scale ranging from 0 (worst general health status) to 100 (best general health status). The EQ-5D has shown good test–retest reliability, as well as good validity [42].

#### Quality of life in alzheimer’s disease (QoL-AD)

The Quality of Life in Alzheimer’s Disease (QoL-AD) developed by Logsdon [43] is a 13-item questionnaire tailored to evaluate changes in mood and physical and cognitive functioning, as well as the quality of relationships in the older adult population with probable AD. Participants are required to rate the 13 items according to their current status using a 4-point scale. Studies have shown that the QoL-AD has good to excellent reliability and validity [44–46].

#### Alzheimer’s disease cooperative study—activities of daily living (ADCS-ADL)

The ADCS-ADL [47] functions as an assessment tool for evaluating the competence of individuals diagnosed with AD across both basic and instrumental activities of daily living. This instrument can be administered in two formats: a caregiver-completed questionnaire format, or a structured interview conducted by a clinician or researcher in collaboration with a caregiver. Responses provided should pertain to the 4 weeks preceding the assessment. The basic activities of daily living [48] items each takes an ADL (e.g., eating) and provides descriptions of the level of competence, with the rater selecting the most appropriate option (e.g. ate without physical help and using a knife; used a fork or spoon but not a knife; used fingers to eat; was usually fed by someone else). The test–retest reliability of the ADCS-ADL is estimated to be high [48].

#### Neuropsychiatric inventory (NPI)

The NPI [49] provides a brief assessment of twelve distinct neuropsychiatric symptoms, including delusions, hallucinations, agitation/aggression, depression, anxiety, euphoria, apathy/indifference, disinhibition, irritability, aberrant motor behaviour, and sleeping problems and eating problems. A judicious screening question, ascertaining the presence or absence of behavioural changes, serves as the initial point of inquiry. Upon an affirmative response to the screening question, the frequency and severity ratings are determined. The frequency is rated on a 4-point Likert scale ranging from 1 (occasionally—less than once per week) to 4 (very frequently—daily or essentially continuously present). The severity is rated as 1 for mild—produces little distress in the patient, 2 for moderate—more disturbing to the patient but can be redirected by the caregiver and 3 for severe—very disturbing to the patient and difficult to redirect. The multiplication of frequency * severity results in a domain score. The total NPI score is calculated by the summation of the twelve domain scores. Inter-rater reliability ranged from 93.6 to 100 (depending on the sub-domain) and test–retest reliability is high [50].

### Plans to promote participant retention and complete follow-up {18b}

Completion of the study trial will result in the reimbursement of a total of 95 euros in vouchers for the participant and 50 euros in vouchers for the (informal) caregiver. In instances of premature termination of participation, the disbursement will be contingent on the number of test days attended by the participant. Provisions for travel-related expenditures will be separately reimbursed, encompassing a rate of 19 euro cents per kilometre and the full coverage of bus and train tickets (second class) adhering to established Dutch guidelines. Participants are called bi-weekly to monitor their well-being, address queries, and ensure ongoing engagement with the study.

In the event of an inability to collect primary outcome data, specifically the 15-VLT, either at baseline or the 6-month follow-up, resulting from circumstances such as discontinuation, the individual will be classified as a study dropout. It is noteworthy that the absence of other data or protocol deviations will not automatically lead to participant dropout. Any protocol deviations that arise will be meticulously recorded in the protocol/legislation deviation log for thorough documentation and subsequent analysis.

### Data management {19}

The data acquisition, entry, and management procedures will be exclusively conducted at Maastricht University. A total of 72 participants will be enrolled in the study, resulting in an approximate data volume of approximately 50 gigabytes. Maastricht University’s standard facilities will be employed for data storage and backup, involving the utilization of a secure server with daily data backups. Stringent adherence to the legal retention period, specifically a minimum of 25 years, and compliance with the General Data Protection Regulation (GDPR) will be observed throughout the data storage process. The study population compromises an elderly population with cognitive impairment, necessitating an extensive battery of tests and paper-and-pencil-based tests and questionnaires. A manual transfer of data from these papers to the eCRF in the CASTOR EDC database will be executed. Before entry into the eCRF, original data will undergo thorough accuracy checks. Instances of missing data will be categorized as measurement failed, not applicable, not asked, asked but unknown, and not performed. A comprehensive data management plan has been formulated by our data management team, consisting of an ICT developer and system administrator from our department, and a Project Specialist from the Clinical Trial Centre Maastricht (CTCM). Their collective expertise spans data management, monitoring, and pharmacovigilance. Adherence to regulatory frameworks, including the Quality Assurance for Research Involving Human Subjects Act, the Code of Conduct for Responsible Use of Human Tissue, and the Medical Treatment Contracts Act will be maintained. The eCRF in CASTOR incorporates built-in range checks for data values. To adhere to Good Clinical Practice (GCP), all modifications are systematically logged, and a justification for altering a field’s value is mandatory, courtesy of the audit feature. The eCRF in CASTOR offers a study progress overview which includes the participant record creation, visit completion, participant status, and visit status. Oversight of the eCRF building is appointed to our project quality specialist from the CTCM, who maintains a list of authorized individuals with editing privileges in CASTOR.

### Confidentiality {27}

To safeguard participant privacy, all collected data will undergo pseudonymization following GDPR guidelines. The code key will be securely managed by the principal investigator. Results will be coded to include the participant’s unique identification code (1 to 81) and the session (e.g. T0 for the first test day (baseline) and T4 for the last one). Printed questionnaires will be labelled with the unique identification code, session number, date, and time of measurement. Personal data, such as informed, consent will be coded (screening number), treated confidentially, and stored separately from other research data. Original data on paper will be stored in a locked cabinet within a secured office with restricted access in the department. Participants will be explicitly informed, through the informed consent process, about authorized individuals with access to their medical and personal data. This list includes the principal investigator, the Health and Youth Care Inspectorate (IGJ), and the study monitor (CTCM). Participants’ data will remain strictly confidential and will not be disclosed to unauthorized third parties, ensuring confidentially throughout the study.

### Plans for collection, laboratory evaluation and storage of biological specimens for molecular analysis in this trial and future use {33}

The storage of human biological material, specifically blood plasma and tear fluid samples, will adhere to a stringent scientific protocol set up with the Biobank of the MUMC + . The samples are securely stored and coded, each assigned a unique subject identification code, in the − 80 °C freezer situated in the ISO-certified Biobank of the MUMC + . The confidentiality of this code is safeguarded by the principal investigator. Upon exclusion from our clinical trial, participants meeting positive exclusion criteria during medical screening undergo immediate pre-analysis destruction of human biological material, such as blood plasma designated for biomarker and exosome collaborations. Participants are informed during the informed consent process that fully included the participant’s human biological material such as tear fluid and blood plasma samples for the collaboration studies, is retained post-conclusion of the ROMEMA study, preserving the potential for future research for a maximum of 5 years. For samples dispatched to external entities for analysis, specifically, blood plasma sent for roflumilast metabolite evaluation, will be pseudonymized. No identifiable participant information is transmitted to the external company. Post-analysis, the company is instructed to destroy the plasma samples. In the context of sub-studies and aforementioned collaborations, the sharing of data with other researchers exclusively involves pseudonymized information. Consistent protocols governing processing and storage timelines are uniformly applied across both the primary study and its associated sub-studies and collaborations.

### Statistical methods

#### Statistical methods for primary and secondary outcomes {20a}

Descriptive statistics for continuous parameters will include sample sizes, means and standard errors of the dependent variables, and covariates for each distinct level combination of factors. For categorical data, summary tables will present counts and percentages. Statistical summaries will be presented by treatment, sequence, or period. The repeated measures, namely the outcome variables of the cognitive assessments and the subjective well-being-related scales, will be analysed using mixed models. The between-subject factor will be the treatment group (three levels: placebo, 50 μg roflumilast, and 100 μg roflumilast). The scores of each task will be entered as the within-subject factor, as well as time (T1, i.e. acute, T2, i.e. 12 weeks, T3, i.e. 24 weeks, T4, i.e. 2-week follow-up). An additional interaction term between the treatment group and time is included to determine whether the different treatment groups will lead to different trajectories over time. Subject covariates included will be sex, age, and education. Additionally, at the end of the study, we will ask each participant which intervention arm they think they have received (placebo, 50 mcg roflumilast, or 100 mcg roflumilast). Because of exclusion and inclusion criteria related to the patient recruitment of the study, the population recruited will be homogenous. Note that groups will not be able to be matched because of the applied randomization block strategy. The significance level will be set at *a* = 0.05.

### Interim analysis {21b}

An interim analysis, methodically embedded within the study protocol, serves as an evaluation of the foundational assumptions underpinning the original design and sample size calculations. The objectives of the interim analysis extend beyond the purview of methodological scrutiny. They encompass the imperative need to inform decisions regarding the continuation of the (UM) patent of roflumilast (US20150051254A1), specifically focusing on its application in the treatment of cognitive impairment. This juncture underscores the translational impact of the study, linking scientific exploration with potential intellectual property considerations.

The execution of the interim analysis necessitates unblinded access to treatment group assignments. This pivotal task will be entrusted to an impartial and independent researcher possessing specialized expertise in repeated measure design. The timing of this interim is aligned with the enrollment trajectory, with the analysis slated to transpire precisely after half of the predetermined total participant cohort have fulfilled their participation commitment.

### Methods for additional analyses (e.g. subgroup analyses) {20b}

Subgroups are defined based on baseline characteristics including sex, age, and education. We will investigate the effect of age in a stratified manner. By stratifying the groups into two age categories, e.g. < 70 and > 70, as well as stratifying the groups in two education categories, i.e. low and high education.

### Methods in analysis to handle protocol non-adherence and any statistical methods to handle missing data {20c}

Advantages of LMM over ANOVA-based repeated measures include that it does not use listwise deletion, as it handles missing observations by using maximum likelihood to estimate missing values conditional on covariates [51].

### Plans to give access to the full protocol, participant-level data and statistical code {31c}

The full protocol, anonymized data set, and statistical code will be available on request after the results of the study have been published.

## Oversight and monitoring

### Composition of the coordinating centre and trial steering committee {5d}

- ***User committee (yearly meetings)***

(research team, representative ZonMw, representative Alzheimer Nederland, representative Hersenstichting, representative client panel Alzheimer Centrum Limburg, health technology assessment officer, business developer, project advisor, and health economist).

- Provide input
- Monitor progress
- Discuss bottlenecks
- Discuss/improve participation and dissemination plan follow-up process to embed the innovation into healthcare

- ***Data management team***

(ICT developer system administrator and project quality specialist).

- Data management plan
- Management access Castor electronic data capturing system (CASTOR)

Trial monitor (Clinical Trial Centre Maastricht; CTCM).

(Project quality specialist).

- Trial monitoring
- General control of data collection
- Verification of source documents and electronic case report forms (eCRFs)
- Controlling compliance with laws and regulations
- Controlling compliance with protocols
- Checking informed consents
- Controlling Trial Master File (TMF)
- Verifying reports of adverse events

Principal investigator (IR).

Executive researcher (NP).

Project leader (FV).

Coordinating researcher (AB).

Coordinating researcher (JP).

Independent researcher (RS).

Research physician (CvL).

Independent medical expert (MvB).

Data management team (RM, WN).

User committee (IR, NP, FV, AB, JP, RH, EH, MF, EB, LP, HB, AS, RS).

Clinical trial monitor (EN).

Design of the study (IR, NP, AB, JP).

Preparation of protocol and revisions (IR, NP, AB, JP).

Ethics committee application (IR, NP).

Study planning (IR, NP).

Recruiting, training, and supervising research assistants (IR, NP).

Responsible for trial master file (IR, NP).

Provide an annual report to ethics committee (IR, NP).

Data verification (IR, NP).

Publication of study reports (IR, NP, AB, JP, FV).

### Composition of the data monitoring committee its role, and reporting structure {21a}

No data monitoring committee DMC was appointed for this trial as this study is classified as a medium risk by the local Medical Research Ethics Committee (MREC), a low burden, and a single-centre study.

### Adverse event reporting and harms {22}

All AEs reported spontaneously by the participant or observed by the investigator or his staff will be recorded. All AEs will be monitored until they have abated, or until a stable situation has been reached. Depending on the event, follow-up may require additional tests or medical procedures as indicated, and/or referral to the general physician or a medical specialist. SAEs need to be reported till the end of the study within the Netherlands, as defined in the protocol. All SAEs will be reported through the web portal ToetsingOnline to the accredited MREC that approved the protocol, within 7 days of first knowledge for SAEs that result in death or are life-threatening followed by a period of maximum 8 days to complete the initial preliminary report. All other SAEs will be reported within a period of a maximum of 15 days after the research team has first knowledge of the SAEs. SAEs that result in death or are life-threatening should be reported expedited. The expedited reporting will occur not later than 7 days after the responsible investigator has first knowledge of the adverse reaction (AR). This is for a preliminary report with another 8 days for completion of the report. Suspected unexpected serious adverse reactions (SUSARs) will be reported through the web portal ToetsingOnline to the MREC: SUSARs that have arisen in the clinical trial that was assessed by the MREC; or SUSARs that have arisen in other clinical trials of the same sponsor and with the same medicinal product, and that could have consequences for the safety of the subjects involved in the clinical trial that was assessed by the MREC.

The remaining SUSARs are recorded in an overview list (line-listing) that will be submitted once every 6 months to the MREC. This line-listing provides an overview of all SUSARs from the study medication, accompanied by a brief report highlighting the main points of concern. The expedited reporting of SUSARs through the web portal Eudravigilance or ToetsingOnline is sufficient as a notification to the competent authority. The investigator will report expedited all SUSARs to the competent authorities in the other Member States, according to the requirements of the Member States. The expedited reporting will occur not later than 15 days after the sponsor has first knowledge of the ARs. For fatal or life-threatening cases, the term will be a maximum of 7 days for a preliminary report with another 8 days for completion of the report. One of the senior researchers not involved in subject testing or data analysis will reveal the individual subject code. The subject will be excluded from the study.

In addition to the expedited reporting of SUSARs, the investigator will submit, once a year throughout the clinical trial, a safety report to the accredited MREC, competent authority, and competent authorities of the concerned Member States. The annual safety report will be combined with the annual progress report. The safety report consists of a list of all suspected (unexpected or expected) SARs, along with an aggregated summary table of all reported SAEs, ordered by organ system, per study, and a report concerning the safety of the subjects, consisting of a complete safety analysis and an evaluation of the balance between the efficacy and the harmfulness of the medicine under investigation.

Potential side effects attributable to the investigational study drug, roflumilast, are reported in the form of the Daxas package leaflet in the patient information sheet. These side effects, extrapolated from the known outcomes associated with the administration of 250 µg and 500 µg roflumilast dosages, serve as a critical informational resource for study participants. Importantly, the informational transparency is accompanied by a candid acknowledgement of the uncertainty regarding the applicability of known side effects to the lower dosages employed in this study, specifically the 50- and 100-µg variants. Acknowledging the nuanced nature of pharmaceutical interventions, the patient information sheet explicitly underscores the proactive surveillance mechanisms instituted to inquire about participants’ emotional and physical well-being. A stringent regimen of regular telephonic engagement has been instituted, empowering participants to promptly communicate any perceived side effects. Additionally, a perpetual availability of a qualified research physician ensures real-time responsiveness to participant inquiries, further amplifying the vigilance inherent in this study. To bolster the ongoing safety evaluation, an iterative process of annual updates to the development safety update report is rigorously adhered to. These updates are dutifully submitted to the evaluation of the MREC.

### Frequency and plans for auditing trial conduct {23}

As this trial falls under the scope of the Dutch Medical Research Involving Human Subjects Act (Dutch: Wet Medisch-Wetenschappelijk Onderzoek Met Mensen; WMO), the CTCM has appointed an independent clinical research monitor to oversee the study. The designated individual monitors whether the study is conducted according to the ICH-GCP guidelines, legislation, and regulations. Collaboratively with the research team, a comprehensive monitoring plan is prepared, in which all details regarding the procedures are stated. This plan is made according to the specific demands of the study and in line with legislation and regulations. The expertise offered by CTCM extends beyond mere monitoring, encompassing advisory roles on intricate matters related to laws and regulations. CTCM, in its oversight capacity, conducts a review of data collection processes, verifying source documents and eCRFs. This review extends to inspecting Informed Consent forms and protocols, ensuring their alignment with regulatory standards. Moreover, CTCM undertakes a vigilant examination of reports pertaining to AEs and complications.

### Plans for communicating important protocol amendments to relevant parties (e.g. trial participants, ethical committees) {25}

Amendments are changes made to the research after approval of the study protocol by the accredited MREC. All amendments will be notified to the MREC that approves the protocol gives a favourable opinion. A “substantial amendment” is defined as an amendment to the terms of the MREC application, or to the protocol or any other supporting documentation, that is likely to affect to a significant degree such as the safety or physical or mental integrity of the subjects of the trial, the scientific value of the trial, the conduct or management of the trial, or the quality or safety of any intervention used in the trial. All substantial amendments will be notified to the MREC and the competent authority. Nonsubstantial amendments do not have to be notified to the accredited MREC and the competent authority but will be recorded and filed by the investigator. If significant amendments are made, participants will be informed and asked to sign a new informed adapted consent. In case of significant amendment, trial registries will be updated.

### Dissemination plans {31a}

All results from this research will be disclosed unreservedly in a scientific paper aimed for publication in a peer-reviewed scientific journal. The list of authors will include all individuals who made substantial contributions to the conception and design, or analysis and interpretation of data, as well as drafting the paper or revising it for important intellectual content. Publication is not restricted or limited in any way by the actual outcome of the study. All results, both negative and positive, will be incorporated in the paper. In addition, the results will be presented at one or more national and/or international scientific conferences either as posters or oral presentations. The participant’s data will not be disclosed to unauthorized third parties, and participant confidentiality will always be maintained. Publication takes place in accordance with the rules in the CCMO statement publication policy.

## Discussion

Building upon our department’s previous investigations involving pronounced age-associated memory impaired/amnestic MCI participants, a prospective delay of 3–5 years in the conversion from MCI to dementia is anticipated [21]. This potential delay holds significant promise, potentially deferring nursing home admissions and thus substantial cost reduction spent on the care of patients diagnosed. Even in cases of eventual conversion, the anticipated benefits of roflumilast, such as cognitive stabilization and neuroprotective effects, are presumed to persist in AD patients. This underscores the potential longevity of positive outcomes, given the average life expectancy of AD dementia patients. In essence, the envisioned efficacy in aspects of daily living has profound implications for cost reduction in care and related expenses.

The investigational utilization of roflumilast, a registered medicinal product, brings forth a consideration of potential AEs and their implication in the context of cognitive function. Our evaluation, drawn from prior studies from our department, predicates that reported AEs are predominantly mild and transient, resolving within 24 h post-intake, even at doses surpassing those tested in the current study. The potential positive outcomes, specifically enhanced or stabilization of cognitive function, neuroprotective effects, and the prospective delay of mild or moderate AD dementia, are strategically weighed against the backdrop of the most prevalent side effects associated with roflumilast. Crucially, the absence of established pharmacological treatments for patients with MCI in Europe underscores the pioneering nature of our study. The study cohort, comprising (geriatric) participants aged 50 to 90, exhibits a lack of specific susceptibilities to roflumilast, reinforcing the scientific rationale for exploration.

To enhance participant safety, a proactive risk reduction approach is instituted, involving regular researcher-patient interactions. A structured communication cadence, including weekly contacts during the initial month and bi-weekly engagements in subsequent months, facilitates the timely documentation of side effects and patient concerns. Weight loss, identified as a common side effect, is vigilantly monitored through weight measurements on each test day. Exclusion criteria, incorporating a BMI threshold of under 18.5, further contribute to refining participant selection and ensuring their well-being.

Access to the Electronic Patient File provides a real-time conduit for the principal investigator and co-investigator to investigate AEs and SAEs for Maastricht University Medical Centre + (MUMC +) participants. This pre-emptive measure empowers the research team with valuable insights before each test day, fostering a proactive stance in ensuring participant safety. Evidence from clinical studies indicates that the majority of patients, post-treatment discontinuation, regain lost weight, allaying concerns related to this common side effect. Importantly, the comprehensive review of clinical studies reveals an absence of reported overdose cases with roflumilast, reinforcing its favourable safety profile.

Finally, the adaptation of exclusion criteria, meticulously tailored to exclude clinical participants at heightened risk of side effects or with diminished therapeutic responsiveness to roflumilast, further exemplifies the study’s commitment to participant safety. In summary, this scientific justification underpins the study’s robust risk mitigation strategies, positioning the investigation of roflumilast for cognitive enhancement as a pioneering and conscientiously conducted endeavor in the realm of pharmacological interventions for cognitive function.

### Trial status

The MREC of the MUMC + and the University of Maastricht granted ethics approval for the 4th version of the protocol on September 10th, 2020. The trial was registered at the European Drug Regulatory Affairs Clinical Trials (EudraCT) register on the 19th of December 2019 and Clinicaltrial.gov (NCT04658654) on the 8th of December 2020. The Central Committee on Research Involving Human Subjects (CCMO) granted approval on the 30th of September 2020. The start of recruitment was November 19th of 2021. Inclusion is currently ongoing. The approximate date when recruitment will be completed is November 2024.

## Data Availability

The datasets used and/or analyzed during the current study are available from the corresponding author upon reasonable request after publication of the results.

## Abbreviations

AD: Alzheimer’s disease
ADAS-Cog: Alzheimer’s Disease Assessment Scale-Cognitive Subscale
ADCS-ADL: Alzheimer’s Disease Cooperative Activities of Daily Living Scale
AE: Adverse event
aMCI: Amnestic mild cognitive impairment
AR: Adverse reaction
BDI-2: Beck Depression Inventory-2
BMI: Body mass index
BNT: Boston Naming Test
CAMP: Cyclic Adenosine Monophosphate
CASTOR: Castor Electronic Data Capturing System
CCMO: Centrale Commissie Mensgebonden Onderzoek
CGMP: Cyclic guanosine monophosphate
COPD: Chronic obstructive pulmonary disease
CDR: Clinical Dementia Rating
CRF: Case report form
CTCM: Clinical Trial Centre Maastricht
CVA: Cerebrovascular accident
ECRF: Electronic case report form
EudraCT: European Drug Regulatory Affairs Clinical Trials
EQ-5D: EuroQol-5 Dimensions
GCP: Good Clinical Practice
GDPR: General Data Protection Regulation
GMP: Good Manufacturing Practice
GP: General practitioner
HADS: Hospital Anxiety and Depression Scale
HIV: Human immunodeficiency virus
HTA: Health technology assessment
IGJ: Inspectie Gezonheidszorg en Jeugd
LDST: Letter Digit Substitution Test
MCI: Mild cognitive impairment
MDMA: Methylenedioxymethamphetamine
MMSE: Mini-Mental State Examination
MREC: Medical Research Ethics Committee
MUMC: Maastricht University Medical Centre
NDE: Neuron-derived exosome
NPH: Normal pressure hydrocephalus
NPI: Neuropsychiatric Inventory
PDE: Phosphodiesterase
PTAU: Phosphorylated tau
ROS: Reactive oxygen species
RCT: Randomized controlled trial
RT: Reaction time
(S)AE: (Serious) adverse event
SD: Standard deviation
SUSAR: Suspected unexpected serious adverse reaction
TIA: Transient ischemic attack
TMF: Trial master file
TMT: Trial Making Test
UM: University of Maastricht
VLT: Verbal Learning Test
WMO: Wet Medischwetenschappelijk Onderzoek met Mensen
QALY: Quality-adjusted life years
QOL-AD: Quality of Life – Alzheimer’s Disease

## References

[1] Marucci G, Buccioni M, Ben DD, Lambertucci C, Volpini R, Amenta F (2021). Efficacy of acetylcholinesterase inhibitors in Alzheimer's disease. Neuropharmacology.

[2] Ibach B, Haen E (2004). Acetylcholinesterase inhibition in Alzheimer's Disease. Curr Pharm Des.

[3] Rammes G, Danysz W, Parsons CG (2008). Pharmacodynamics of memantine: an update. Curr Neuropharmacol.

[4] Karakaya T, Fusser F, Schroder J, Pantel J (2013). Pharmacological Treatment of Mild Cognitive Impairment as a Prodromal Syndrome of Alzheimer s Disease. Curr Neuropharmacol.

[5] Sokolow S, Li X, Chen L, Taylor KD, Rotter JI, Rissman RA (2017). Deleterious Effect of Butyrylcholinesterase K-Variant in Donepezil Treatment of Mild Cognitive Impairment. J Alzheimers Dis.

[6] Heckman PR, Wouters C, Prickaerts J (2015). Phosphodiesterase inhibitors as a target for cognition enhancement in aging and Alzheimer's disease: a translational overview. Curr Pharm Des.

[7] Bender AT, Beavo JA (2006). Cyclic nucleotide phosphodiesterases: molecular regulation to clinical use. Pharmacol Rev.

[8] McLachlan CS, Chen ML, Lynex CN, Goh DL, Brenner S, Tay SK (2007). Changes in PDE4D isoforms in the hippocampus of a patient with advanced Alzheimer disease. Arch Neurol.

[9] Ugarte A, Gil-Bea F, Garcia-Barroso C, Cedazo-Minguez A, Ramirez MJ, Franco R (2015). Decreased levels of guanosine 3', 5'-monophosphate (cGMP) in cerebrospinal fluid (CSF) are associated with cognitive decline and amyloid pathology in Alzheimer's disease. Neuropathol Appl Neurobiol.

[10] Rutten K, Basile JL, Prickaerts J, Blokland A, Vivian JA (2008). Selective PDE inhibitors rolipram and sildenafil improve object retrieval performance in adult cynomolgus macaques. Psychopharmacology.

[11] Sutcliffe JS, Beaumont V, Watson JM, Chew CS, Beconi M, Hutcheson DM (2014). Efficacy of selective PDE4D negative allosteric modulators in the object retrieval task in female cynomolgus monkeys (Macaca fascicularis). PLoS ONE.

[12] Myeku N, Clelland CL, Emrani S, Kukushkin NV, Yu WH, Goldberg AL (2016). Tau-driven 26S proteasome impairment and cognitive dysfunction can be prevented early in disease by activating cAMP-PKA signaling. Nat Med.

[13] Blokland A, Menniti FS, Prickaerts J (2012). PDE inhibition and cognition enhancement. Expert Opin Ther Pat.

[14] Prickaerts J, Heckman PRA, Blokland A (2017). Investigational phosphodiesterase inhibitors in phase I and phase II clinical trials for Alzheimer's disease. Expert Opin Investig Drugs.

[15] Puhan M (2011). Phosphodiesterase 4 inhibitors for chronic obstructive pulmonary disease. Cochrane Database Syst Rev.

[16] Blokland A, Van Duinen MA, Sambeth A, Heckman PRA, Tsai M, Lahu G (2019). Acute treatment with the PDE4 inhibitor roflumilast improves verbal word memory in healthy old individuals: a double-blind placebo-controlled study. Neurobiol Aging.

[17] Anderson ND (2019). State of the science on mild cognitive impairment (MCI). CNS Spectr.

[18] Bonner-Jackson A, Mahmoud S, Miller J, Banks SJ (2015). Verbal and non-verbal memory and hippocampal volumes in a memory clinic population. Alzheimers Res Ther.

[19] McPhee I, Cochran S, Houslay MD (2001). The novel long PDE4A10 cyclic AMP phosphodiesterase shows a pattern of expression within brain that is distinct from the long PDE4A5 and short PDE4A1 isoforms. Cell Signal.

[20] Fennema-Notestine C, McEvoy LK, Hagler  DJ, Jacobson MW, Dale AM (2009). The Alzheimer's Disease Neuroimaging I. Structural neuroimaging in the detection and prognosis of pre-clinical and early AD. Behav Neurol.

[21] Hamel R, Kohler S, Sistermans N, Koene T, Pijnenburg Y, van der Flier W (2015). The trajectory of cognitive decline in the pre-dementia phase in memory clinic visitors: findings from the 4C-MCI study. Psychol Med.

[22] Roda M, Ciavarella C, Giannaccare G, Versura P (2020). Biomarkers in Tears and Ocular Surface: A Window for Neurodegenerative Diseases. Eye Contact Lens.

[23] McKhann GM, Knopman DS, Chertkow H, Hyman BT, Jack CR, Kawas CH (2011). The diagnosis of dementia due to Alzheimer's disease: recommendations from the National Institute on Aging-Alzheimer's Association workgroups on diagnostic guidelines for Alzheimer's disease. Alzheimers Dement.

[24] Van Duinen MA, Sambeth A, Heckman PRA, Smit S, Tsai M, Lahu G (2018). Acute administration of roflumilast enhances immediate recall of verbal word memory in healthy young adults. Neuropharmacology.

[25] Welton RF, Han BX, Stockli MP, Murray SN, Pennisi TR, Stinson C (2020). Installation and commissioning of the ion source systems for the new spallation neutron source 2.5 MeV injector. Rev Sci Instrum.

[26] Smith D, Lovell J, Weller C, Kennedy B, Winbolt M, Young C (2017). A systematic review of medication non-adherence in persons with dementia or cognitive impairment. PLoS ONE.

[27] Van der Elst W, van Boxtel MP, van Breukelen GJ, Jolles J (2005). Rey's verbal learning test: normative data for 1855 healthy participants aged 24–81 years and the influence of age, sex, education, and mode of presentation. J Int Neuropsychol Soc.

[28] Garces Molina FJ, Royo Garcia A, Hernandez Perez G, Pinilla B, Pastor Gomez-Cornejo L, Portugal AJ (2000). Cytomegalovirus diarrhea as a diagnostic index for AIDS. An Med Interna.

[29] Brand N, Jolles J (1985). Learning and retrieval rate of words presented auditorily and visually. J Gen Psychol.

[30] Kok R, Verhey F (2002). Gestandaardiseerde MMSE.

[31] Baek MJ, Kim K, Park YH, Kim S (2016). The Validity and Reliability of the Mini-Mental State Examination-2 for Detecting Mild Cognitive Impairment and Alzheimer's Disease in a Korean Population. PLoS ONE.

[32] Reitan RM (1958). Validity of the Trail Making Test as an indicator of organic brain damage. Percept Mot Skills.

[33] Davis HP, Small SA, Stern Y, Mayeux R, Feldstein SN, Keller FR (2003). Acquisition, recall, and forgetting of verbal information in long-term memory by young, middle-aged, and elderly individuals. Cortex.

[34] van der Elst W, van Boxtel MP, van Breukelen GJ, Jolles J (2006). The Letter Digit Substitution Test: normative data for 1,858 healthy participants aged 24–81 from the Maastricht Aging Study (MAAS): influence of age, education, and sex. J Clin Exp Neuropsychol.

[35] Mack WJ, Freed DM, Williams BW, Henderson VW (1992). Boston Naming Test: shortened versions for use in Alzheimer's disease. J Gerontol.

[36] Kueper JK, Speechley M, Montero-Odasso M (2018). The Alzheimer's Disease Assessment Scale-Cognitive Subscale (ADAS-Cog): Modifications and Responsiveness in Pre-Dementia Populations. A Narrative Review J Alzheimers Dis.

[37] Rosen WG, Mohs RC, Davis KL (1984). A new rating scale for Alzheimer's disease. Am J Psychiatry.

[38] Kirwan CB, Stark CE (2007). Overcoming interference: an fMRI investigation of pattern separation in the medial temporal lobe. Learn Mem.

[39] Zigmond AS, Snaith RP (1983). The hospital anxiety and depression scale. Acta Psychiatr Scand.

[40] Bjelland I, Dahl AA, Haug TT, Neckelmann D (2002). The validity of the Hospital Anxiety and Depression Scale. An updated literature review. J Psychosom Res.

[41] Herdman M, Gudex C, Lloyd A, Janssen M, Kind P, Parkin D (2011). Development and preliminary testing of the new five-level version of EQ-5D (EQ-5D-5L). Qual Life Res.

[42] Feng YS, Kohlmann T, Janssen MF, Buchholz I (2021). Psychometric properties of the EQ-5D-5L: a systematic review of the literature. Qual Life Res.

[43] Logsdon RG, Teri L, Weiner MF, Gibbons LE, Raskind M, Peskind E (1999). Assessment of agitation in Alzheimer's disease: the agitated behavior in dementia scale. Alzheimer's Disease Cooperative Study. J Am Geriatr Soc.

[44] Bárrios HSG. Adaptação cultural e linguística e validação do instrumento QOL-AD para Portugal 2013.

[45] Buasi N, Permsuwan U (2014). Validation of the Thai QOL-AD version in Alzheimer's patients and caregivers. Australas Med J.

[46] Thorgrimsen L, Selwood A, Spector A, Royan L, de Madariaga LM, Woods RT (2003). Whose quality of life is it anyway? The validity and reliability of the Quality of Life-Alzheimer's Disease (QoL-AD) scale. Alzheimer Dis Assoc Disord.

[47] Galasko D, Bennett D, Sano M, Ernesto C, Thomas R, Grundman M (1997). An inventory to assess activities of daily living for clinical trials in Alzheimer's disease. The Alzheimer's Disease Cooperative Study. Alzheimer Dis Assoc Disord.

[48] Weyer G, Erzigkeit H, Kanowski S, Ihl R, Hadler D (1997). Alzheimer's Disease Assessment Scale: reliability and validity in a multicenter clinical trial. Int Psychogeriatr.

[49] Cummings JL, Mega M, Gray K, Rosenberg-Thompson S, Carusi DA, Gornbein J (1994). The Neuropsychiatric Inventory: comprehensive assessment of psychopathology in dementia. Neurology.

[50] Cummings JL (1997). The Neuropsychiatric Inventory: assessing psychopathology in dementia patients. Neurology.

[51] Krueger C, Tian L (2004). A comparison of the general linear mixed model and repeated measures ANOVA using a dataset with multiple missing data points. Biol Res Nurs.

